# Differential Interaction of Antimicrobial Peptides with Lipid Structures Studied by Coarse-Grained Molecular Dynamics Simulations

**DOI:** 10.3390/molecules22101775

**Published:** 2017-10-20

**Authors:** Galo E. Balatti, Ernesto E. Ambroggio, Gerardo D. Fidelio, M. Florencia Martini, Mónica Pickholz

**Affiliations:** 1 Departamento de Física, Facultad de Ciencias Exactas y Naturales, CONICET-Universidad de Buenos Aires, IFIBA, Buenos Aires C1428BFA, Argentina; gbalatti@df.uba.ar; 2 Centro de Investigaciones en Química Biológica de Córdoba (CIQUIBIC), Departamento de Química Biológica “Dr. Ranwel Caputto”, Facultad de Ciencias Químicas, Universidad Nacional de Córdoba, Córdoba X500HUA, Argentina; ambroggioernesto@gmail.com (E.E.A.); gerardo.fidelio@gmail.com (G.D.F.); 3 Departamento de Farmacología, Instituto de la Química y Metabolismo del Fármaco (IQUIMIFA), Facultad de Farmacia y Bioquímica, Cátedra de Química Medicinal, CONICET-Universidad de Buenos Aires, Buenos Aires C1113AAD, Argentina; flormartini1@gmail.com

**Keywords:** maculatin, aurein, helicoidal peptides, lipid bilayers, molecular dynamics, coarse-grain

## Abstract

In this work; we investigated the differential interaction of amphiphilic antimicrobial peptides with 1-palmitoyl-2-oleoyl-sn-glycero-3-phosphocholine (POPC) lipid structures by means of extensive molecular dynamics simulations. By using a coarse-grained (CG) model within the MARTINI force field; we simulated the peptide–lipid system from three different initial configurations: (a) peptides in water in the presence of a pre-equilibrated lipid bilayer; (b) peptides inside the hydrophobic core of the membrane; and (c) random configurations that allow self-assembled molecular structures. This last approach allowed us to sample the structural space of the systems and consider cooperative effects. The peptides used in our simulations are aurein 1.2 and maculatin 1.1; two well-known antimicrobial peptides from the Australian tree frogs; and molecules that present different membrane-perturbing behaviors. Our results showed differential behaviors for each type of peptide seen in a different organization that could guide a molecular interpretation of the experimental data. While both peptides are capable of forming membrane aggregates; the aurein 1.2 ones have a pore-like structure and exhibit a higher level of organization than those conformed by maculatin 1.1. Furthermore; maculatin 1.1 has a strong tendency to form clusters and induce curvature at low peptide–lipid ratios. The exploration of the possible lipid–peptide structures; as the one carried out here; could be a good tool for recognizing specific configurations that should be further studied with more sophisticated methodologies.

## 1. Introduction

Antimicrobial peptides (AMPs) are an essential part of the innate immune system that is found in the three domains of life. They are the first line of defense against external agents [1]. The AMPs act in response to different disturbing biological processes, such as wall synthesis or enzyme activity [2], and a large group of them interact with lipid bilayers, disrupting the membrane integrity and even reaching, in some cases, membrane lysis [3]. The AMPs’ targets are broad, acting among bacteria, fungi, viruses, and eukaryotic parasites [4]. For this reason, AMPs are good candidates to deal with the actual antimicrobial resistance problem [5]. Furthermore, ongoing investigations are focused on the possible applications of AMPs as anticancer drugs [6,7], antibiofilms [8], and as immunomodulators [9,10]. Nevertheless, problems with sensitivity to proteolytic degradation, cell selectivity, and oral absorption raise some pharmaceutical challenges. In any case, the potential use of them as pharmacological drugs deserves exhaustive knowledge of AMP activity at the molecular level [2,11].

Two main molecular mechanisms were proposed for lytic AMPs in membranes [12]. The first of these proposed mechanisms is the pore formation. In this case, after membrane binding, peptides can stabilize such a structure so that it is able to communicate between the inner and outer compartments of the membrane, allowing water and aqueous solutes to cross through it. The known type of pores described at the moment for AMPs are: (1) a “barrel-stave” structure, with the lipid heads remaining in the same interfacial orientation; and (2) a “toroidal” structure, where the head groups of the lipids are oriented along the pore. The second mode is the “carpet” mechanism. In this case, the AMPs would act as a surfactant, disrupting the membrane bilayer and inducing membrane solubilization.

There is a particular type of amphipathic AMPs, the cationic α-helical peptides [13]. Their cationic character favors the interaction of the peptides with the anionic lipids of the organism's membrane with which they interact. In this sense, amphiphilicity and charge make AMPs perfect active membrane molecules that, can affect, for instance, permeability, membrane curvature, and thickness when inserted into the lipid bilayer [14,15].

The behavior of helical AMPs at a molecular level, and the details of their interactions with lipids bilayers, have been successfully studied by classical atomistic molecular dynamics (MD) simulations [16]. However, this kind of system has size and time scale limitations that prevent its use in studying collective effects (e.g., a peptide pore). These restrictions can be solved by the use of simplified coarse-grained (CG) models that nowadays have reached good agreement with atomically detailed simulations. Particularly, the MARTINI CG model has been used in several studies for the simulations of AMPs interacting with lipid bilayers, and has shown the formation of pores in some cases [17,18,19,20,21,22,23,24,25].

The goal of this work is to understand the structural and dynamic properties of two of these helical cationic types of AMPs within lipid membranes. The selected AMPs are aurein 1.2 [6] and maculatin 1.1 [26], which are peptides found in the skin of Australian tree frogs. These peptides showed remarkable differences in their lytic mechanism as well as the peptide concentration required for it. In this scene, when 1-palmitoyl-2-oleoyl-sn-glycero-3-phosphocholine (POPC) giant unillamelar vesicles GUVs were exposed to maculatin 1.1, experimental evidence of pore-forming activity was reported [27]. On the other hand, when POPC GUVs were exposed to aurein 1.2, it was suggested that the “carpet” lytic mechanism acted against those liposomes [27]. However, at this stage, there are no insights on the specific molecular mechanism developed by these AMPs.

In this direction, here we report extensive MD simulations based on the MARTINI CG model, in order to shed light on the differential interaction of these peptides with lipid structures. Since the activity of maculatin 1.1 and aurein 1.2 have already been studied against POPC liposomes, we have chosen phosphatidylcholine (PC) as the model lipid for the simulations. Specifically, to investigate the peptide–lipid interaction mechanism, we have started the simulations from different initial conditions and explored the system’s size and concentration effects. Our in silico experiments are an attempt to contribute to the understanding of the differential effects of both peptides when interacting with lipid bilayers. We base our discussion on the relevance of the knowledge of the activity of AMPs at the molecular level to understand the behavior of similar peptides. The three initial conditions allow us to broadly explore the configuration space.

## 2. Results and Discussion

### 2.1. Aurein 1.2 and Maculatin 1.1 Structural Overview

**Figure 1:**
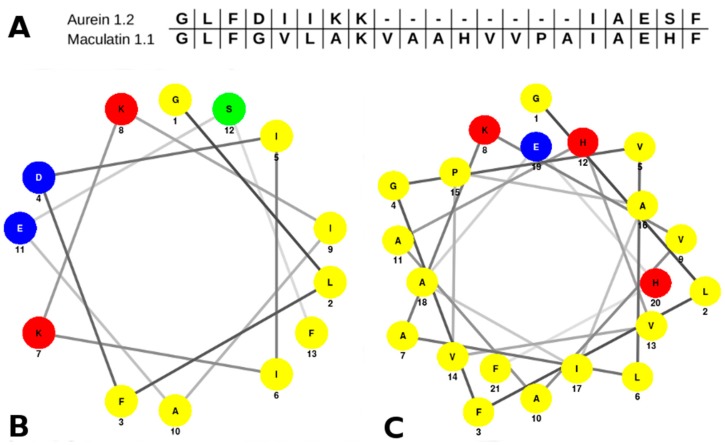
Pairwise alignment (**A**) of aurein 1.2 and maculatin 1.1 sequences, and helical wheel projections for aurein 1.2 (**B**) and maculatin 1.1 (**C**). Residues are colored according to their chemical character as follows: acidic (blue), basic (red), non-polar (yellow), and polar (green).

Both aurein 1.2 and maculatin 1.1 share common characteristics, which are described in most of the helicoidal antimicrobial peptides. They are short cationic alpha-helices, with an amphipathic character and a large percentage of hydrophobic residues. Aurein 1.2 has a shorter sequence than maculatin 1.1. It has 13 residues (GLFDIIKKIAESF-NH2), while maculatin has 21 residues (GLFGVLAKVAAHVVPAIAEHF-NH2). The sequence similarity between both peptides is remarkable at the N-Terminal (NT)/C-Terminal (CT) endings, as is clear in the pairwise sequence alignment (Figure 1A). The differences between the sequences are represented by a wide gap at the center, and most of the mismatch residues are highly hydrophobic; hence, there is a higher global hydrophilicity in aurein. We have also estimated the average hydrophilicity using the Hopp and Wood’s scale [28], which is 0.0 for aurein and −0.7 for maculatin. The ratio of hydrophilic residues over the total amount is higher in aurein (38%) than in maculatin (10%).

On the other hand, the amphipathic character of a helical peptide can be quantitatively represented by the hydrophobic moment μH, since amphiphilicity plays a key role for interfacial membrane binding and partition [29]. From our analysis, we observe that both peptides have very similar μH values: 6.77 for aurein, and 6.8 for maculatin.

Nevertheless, while the hydrophobic moment is virtually the same in both peptides, the distribution of amino acids along the α-helix is slightly different. The helical wheel plot depicted in Figure 1B,C reveals two well-defined distributions: the polar and non-polar residues for both peptides, conforming to two different surfaces of the helix. However, due the presence of many hydrophobic residues in the maculatin sequence, the polar surface of the maculatin helix is small compared with the non-polar surface. In the case of aurein, both surfaces are similar. In addition, the presence of a central proline in the maculatin helix structure induces the formation of a kink. It was suggested that this structural feature may provide an optimal amphiphilic configuration and, in fact, the mutation of PRO-15 to alanine or glycine (more flexible residues) reduces the lytic action of the maculatin mutants exposed to anionic vesicles [27].

### 2.2. MD Simulations

Aiming to understand the lipid peptide interactions, we carried out extensive MD simulations using a coarse-grain model (see the Methods section for more details). We have chosen 1-palmitoyl-2-oleoyl-sn-glycero-3-phosphatidyl-choline (POPC) as the lipid model. The phosphatidylcholine lipid types are the most abundant among mammal endothelial cell membranes, and are broadly used for biomimetic studies. In Table 1, we summarized the different cases simulated in this work, together with the system size and simulation time length.

**Figure 2:**
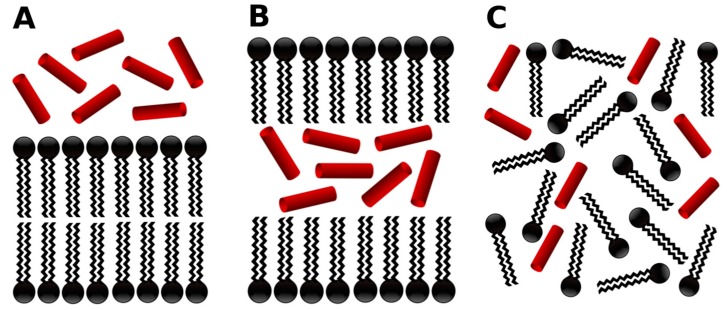
Schematic representation of the three initial simulated states: soluble peptides near to the lipid bilayer interface (**A**); peptides at the hydrophobic core region of the membrane (**B**); and an all-random distribution of the molecules in the simulation box (**C**).

The systems were built up from three main initial peptide–lipid arrangements, as follows, and depicted in Figure 2:

*-out* systems: peptides were placed homogeneously distributed across the XY plane, 3–5 nm away from a pre-assembled lipid bilayer (Figure 2A).
*-in* systems: peptides were placed inside the hydrophobic core of a pre-assembled lipid bilayer (Figure 2B).
*-self* systems: molecules were randomly distributed in the simulation box. We have followed three different randomization configurations in order to validate the strategy (as discussed below, and illustrated in Figure 2C).

At the same time, for the *-self* system, three initial configurations of 50 peptide molecules with 1000 POPC molecules were raised:

- *Self-A*, a homogeneous distribution of molecules, where peptides and lipids were uniformly distributed with no preferential orientation or proximity.
- *Self-B*, with peptides closely located and, thus, prioritizing the peptide–peptide proximity.
- *Self-C*, where the lipid–lipid interaction was favored following the same as for *self-B*.

Cooperative effects play an important role on the peptide pore formation in lipid structures. The mechanism of pore formation of lytic peptides is thought to involve binding to the membrane surface, followed by insertion at threshold levels of bound peptides [30]. In order to bypass this problem and obtain insights on the complex supramolecular arrangements, we carried out the self-assembly simulations. We are aware that, in these kind of simulations, many factors could direct the final structure, such as pressure, temperature, the ratio between amphiphiles and water, etc. Nevertheless, here we choose the same lipid–water–peptide ratio than for a pre-assembled systems, as well as the simulation conditions.

#### Aurein/POPC Simulations

**Figure 3:**
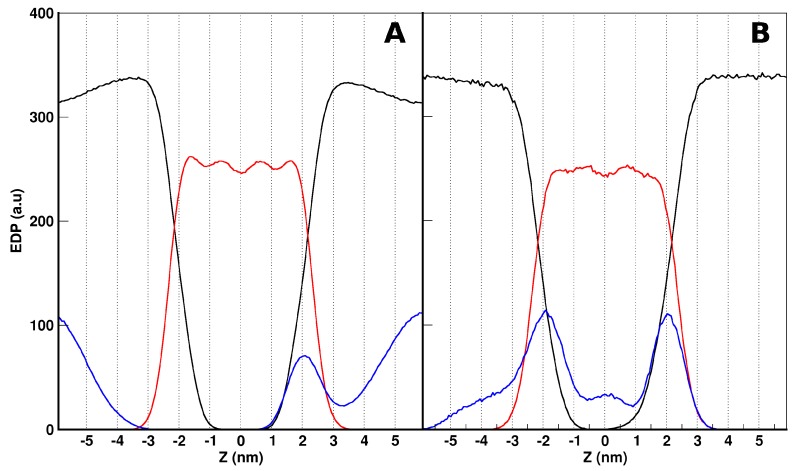
Electronic density profile (EDP) for aurein systems of the -*out* (**A**) and -*in* (**B**) cases. The aurein distribution, shown in blue, as magnified 10×. Water and 1-palmitoyl-2-oleoyl-sn-glycero-3-phosphocholine (POPC) distributions are depicted in black and red, respectively.

In order to obtain an idea of the behavior of the peptides in the above-mentioned simulation systems, we firstly analyzed the overall organization of *the aurein-in* and *aurein-out* configurations. We have calculated the electron density profiles (EDP) normal to the bilayer, averaged over the last 400 ns (ensuring convergence). In Figure 3A,B, we show the EDPs of the different components of the two systems (*-out* and *-in* at the top and bottom, respectively), as a function of the Z coordinate. Z = 0 corresponds to the bilayer center. For both cases, we can see a symmetric distribution of the lipids organized in a bilayer. Additionally, the water distributions drop from a water bulk to the lipid head interface with no access to the bilayer core.

**Figure 4:**
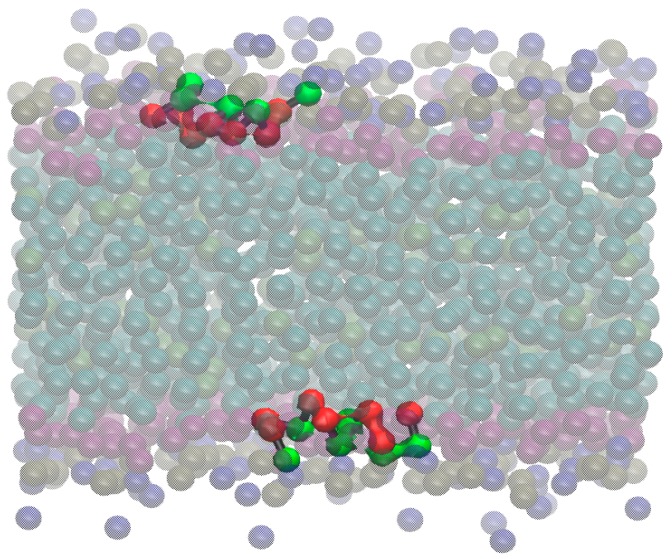
The *small-out* aurein system. Isolated molecules of aurein quickly interact with the lipid–water interface, laying over it with a well-defined orientation: hydrophilic residues (green) are facing the water, and the non-polar ones are orientated to the bilayer core. This kind of behavior was present in all of the simulations with aurein when the molecules are isolated.

With respect to the aurein EDP distribution, we found different results depending on the initial conditions. For *aurein-out* (Figure 3A), the peptides showed two well-defined populations, with no access to the bilayer core. One population is present mainly at the water phase, and the other is present in one of the lipid–water interfaces. Looking further to the aurein peptides at the interface, we found that most of them strongly interact with the lipid head groups, showing a preferential orientation. For simplicity, in Figure 4, we illustrated this behavior through a snapshot of the *small-out* system. In this picture, it is possible to see that single peptide molecules are laid at the lipid–water interface with a defined orientation. The non-polar helix face of aurein (depicted in red) turns into the interior of the membrane, and the polar one (green) are exposed to the solvent. This behavior is also found for the isolated peptides of the *aurein-out*. The other peptide population observed in the *aurein-out* system (Figure 3A) corresponds to a large cluster of aggregated aurein peptides in the water phase. Nearly 80% of the aureins are involved in this cluster. Moreover, this cluster interacts with the POPC bilayer interface. The trajectory of visual inspections show a starting bundle formed in a few nanoseconds acting as a nucleation center of isolated peptides located in the interface and remaining adsorbed during the whole simulation.

It is important to remark that, from the *-out* condition, peptides could not access the hydrophobic region of the bilayer, at least during the simulation time scale. In this direction, we decided to explore the situation where the peptides were originally placed inside the hydrophobic core of the lipid bilayer (*-in* case), without paying attention to the mechanism of accessing this region. From this condition, we can see different behaviors (Figure 3B): on average, peptides are distributed along all regions, with two main peaks at both lipid–water interfaces. Here, aurein was also found at the water and bilayer core regions. About half of the aureins are isolated and laid at the interface with the previously described orientation, while the other half of the molecules are aggregated inside the membrane core and all along the bilayer. The aggregated structure of aureins crosses the membrane, connecting both leaflets and forming a hydrophilic channel inside with water flux through it. We illustrated the structure of this behavior appealing to a small bilayer configuration (*small-in* in Table 1).

**Figure 5:**
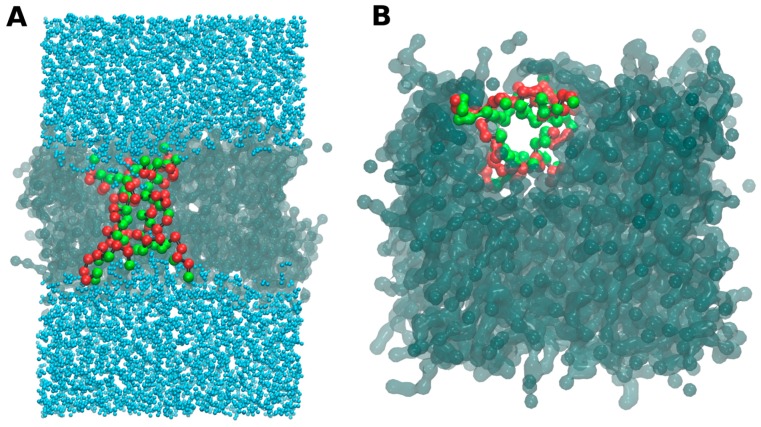
*Small-in* aurein system and the pore-like structure. Snapshots from a frontal plane (**A**) and over the XY bilayer plane (**B**). Green balls represent polar amino acids, while the red balls represent the non-polar amino acids.

In Figure 5, we show a snapshot of this bilayer where the peptides are organized in a pore-like structure with a preferential orientation that follows their amphipathic character: the interior of the channel is a wall of polar amino acids, with the non-polar residues of the helices facing the tails of the lipids in the bilayer. This feature, together with a specific channel size, can lead to an important increase of the water permeability though the membrane when these kinds of structures are formed, despite the CG water bead. 

**Figure 6:**
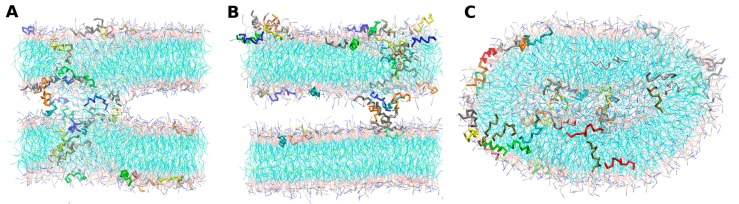
Representative snapshots of Aurein-self simulations: *Self-A* (**A**), *Self-B* (**B**) and *Self-C* (**C**) cases, respectively.

Indeed, there are water molecules inside the channel all along the simulation time (see Figure S1 in the Supplementary Material). Furthermore, to the described organization, this structure shows a special aurein organization. In this direction, in Figure 6A, we show the EDP for the NT and CT terminal groups of the *aurein-in* system. In this figure, we can observe asymmetric distributions and a different behavior between the groups. NT terminals are found at the lipid–water interface region, while the CT terminals face the bilayer core. In order to further analyze this preferential orientation of the peptides, we defined a vector **v** going from CT to NT beads, and we measured the angles formed by **v** with the normal to the bilayer (*z*-axis) all along the simulation time. We have found two preferential angles in ~70° and ~115°, as can be appreciated in the angle frequency histogram (Figure 6B). Both angles represent the same relative orientation with respect to the bilayer plane. In this way, the orientation of peptides inside the pore structure presents angles of approximately 20°–25° with the plane of the bilayer.

**Figure 7:**
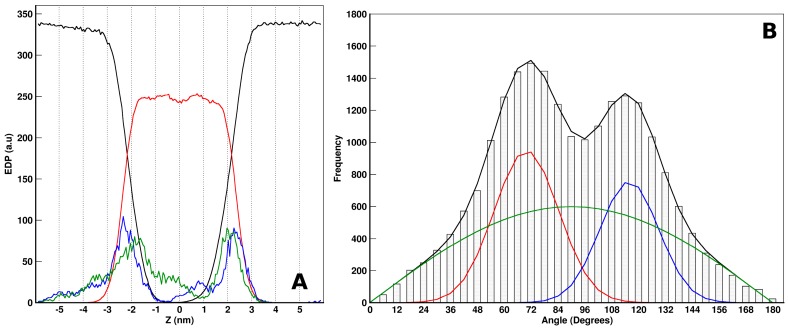
Electron density profiles (EDP) system profile (**A**) of *aurein-in* case, calculated over the last 400 ns of the run. Amino terminals (blue) and carboxyl terminals (green) show preferential position at the *Z*-axis. Water and POPC molecules are depicted in black and red, respectively. Cumulative distribution function (**B**) for the aurein peptide angle with respect to the *Z*-axis. A non-linear regression with a three-term equation was performed. Two angle populations (red and blue lines) and a third of randomly-distributed angles (green) were identified.

The *-in* simulations were performed with the aim to bypass the barrier of the lipid–water interface peptide penetration. An alternative way to achieve this is to perform self-assembly simulations. In this direction, we started from three different randomly-mixed systems that have already been described in Section 2.2. Regardless of the initial configuration, all of the cases derived to the formation of well-defined POPC lipid bilayers, as shown in Figure 7. *Self-A* and *self-B* cases lead to double bilayer structures containing peptide aggregates.

The *self-C* case reached the formation of a liposome-like structure with a non-spherical, but ovoid, shape, with little water inside. In *self-A*, aurein molecules are aggregated, communicating the four leaflets through one continuous superstructure that share all of the structural characteristics obtained in the *-in* cases described above. Despite this fact, the *self-B* channel is wider, probably due to the initial proximity between peptide molecules. Additionally, peptide molecules that are not involved in pore structures conform to an alternative peptide population that follows the same behavior of the described aurein isolated molecules in the previous simulations (self-assemblies and also *-out* and *-in* cases). From the *self-C* condition, we did not observe any pore-like structures during the simulation run: peptides cannot lead to a structure inside the membranes. In this case, a group of aureins follows the behavior of *-out* isolated molecules, through binding to the lipid–water interface with a facial orientation, while the other group conforms to a small cluster inside the aqueous core of the liposome.

### 2.3. MD Simulations: Maculatin/POPC Systems

**Figure 8:**
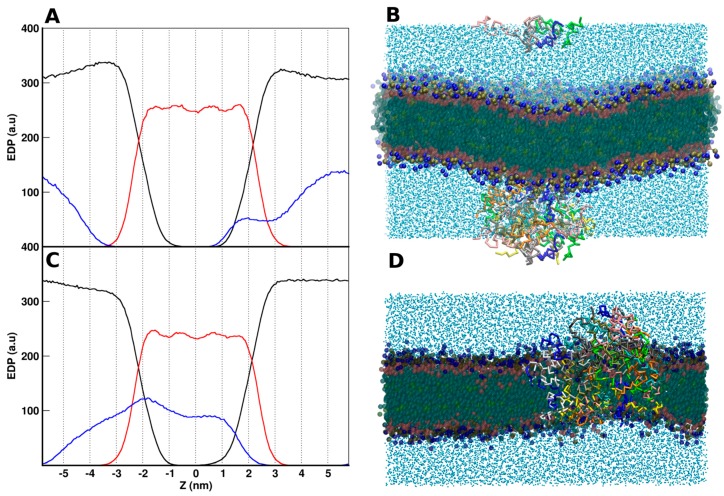
Electron density profiles as function of Z. Lipid, maculatin, and water are shown in red, blue, and black, respectively. (**A**) maculatin-out (**C**) maculatin-in. Representative Snaphots of the systems are shown in (**B**) maculatin-out and (**D**) maculatin-in.

In this section, we show the main results obtained from the study of the maculatin interactions with POPC lipids in different simulated conditions, as discussed for aurein. The EDPs normal to the lipid bilayer of the main system components for *maculatin-out* and *maculatin-in* systems are shown in Figure 8A,C, respectively. The POPC distribution shows a bilayer organization for both cases, with hydrated lipid heads. However, the maculatin distribution presents differences between them: in the *maculatin-out* system, the peptide is mainly localized in water and at the lipid interface with no access to the hydrophobic region of the bilayer, while *maculatin-in* shows an asymmetrical peptide distribution in the whole bilayer and water. Visual inspection of the trajectories gives us more clues on these distributions with a common feature: the strong tendency of maculatins to aggregate themselves. In both cases, most peptides are involved in a big cluster. The strong maculatin aggregation was also observed by atomistic MD simulations when peptides were placed in water (data not shown).

In the *maculatin-out* case, the peptides reached the bilayer interface, and formed a large unique cluster in a few nanoseconds, This cluster remains adsorbed at the lipid–water interface during the whole simulation time, as illustrated in Figure 8B. The peptide cluster cannot penetrate the lipid–water interface in order to access the bilayer core. However, it markedly perturbs the bilayer conformation, changing its curvature. In addition, when maculatins are far enough between each other, they bound to the lipid–water interface, similar to what was observed for aurein (small-out case).

**Figure 9:**
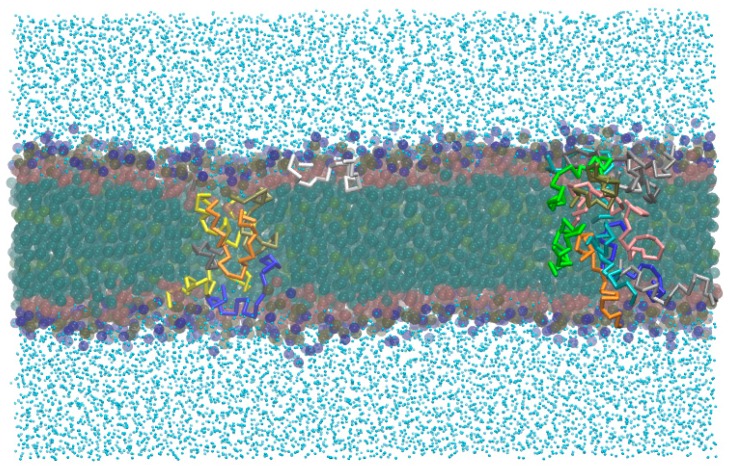
Snapshot of the *in-B* system: two maculatin clusters were observed.

The *maculatin-in* peptides also aggregate into a peptide cluster, but the peculiarity in this case is that this cluster crosses the bilayer, forming a pore-like structure together with a portion of it protruding to the lipid interface and bulk water (Figure 8D). In order to explore whether the presence of this cluster was associated with the particular lipid–peptide ratio used for this case, we included an extra example with just 20 peptides and the same amount of lipids, starting with the peptides distributed inside the bilayer (named as *in-B* in Table 1). In this case, two aggregates were formed in a few nanoseconds, as exemplified in the snapshot in Figure 9, and they remain stable during the rest of the simulation run. The structure of these aggregates was similar to the one observed in the *in-A* system, as discussed below. Unlike the findings in the *aurein-in* case, these clusters were not very permeable to water molecules, and did not show an organized structure. The small hydrophilic surface of maculatin helix may reduce the potential hydrophilic interactions of water with peptides in order to access the bilayer core. In fact, few water molecules were found at the hydrophobic core of the maculatin aggregates. A further view of this kind of structure depicting all of these characteristics is included in the Supplementary Material (Figure S2). The peptides involved in the maculatin aggregates show a low level of internal organization. Neither the orientation of terminals nor the particular angles exhibited in the aurein structures were observed in those structures of maculatin. This was also confirmed by looking at the different groups’ EDPs (see Supporting Material, Figure S3).

**Figure 10:**
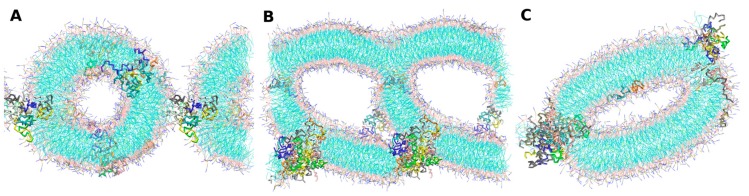
*Maculatin-self* simulations *self-A*, *self-B*, and *self-C* ((**A**)–(**C**), respectively).Peptide chains are depicted in different colors. Water was removed for visualization purpose.

We also carried out self-assembly simulations for maculatin, as described for aurein. The simulations were started from three different random mixes: *self-A*, *self-B*, and *self-C* (see Table 1). Here, the three cases led to similar results: the formation of POPC bilayer structures that were stable during the whole simulation run. Interestingly, in the presence of maculatin peptides, the lipids adopt a cylindrical symmetry structure. In Figure 10, we show a representative snapshot for each of the three *-self* cases. The behavior of maculatin molecules follows the tendency observed in *maculatin-in* systems: high levels of aggregation forming bunch structures. These structures share the same characteristics of the aggregates described above: peptide clustering that crosses along the bilayer normally, with no particular organization of specific groups, together with a low permeation of water molecules. An overall effect we would like to remark is that the presence of maculatin promotes the increase of membrane curvature. This seems to be a characteristic of maculatin that is more pronounced in the self-assembly cases, which strengthens the use of this type of procedure.

### 2.4. Peptides Differential Behavior

In this section, we compared the results found for maculatin and aurein peptides in their interaction with lipid structures. Both peptides share several structural features that distinguish the family of antimicrobial alpha-helical peptides, as they are similar to each other in net charge and hydrophobic moment, while showing differences in length and global hydrophilicity.

The sequence alignment between both peptides (Figure 1A) shows a high percentage of sequence similarity at the peptide endings with a wide gap in the middle, which is composed of hydrophobic residues (except for Hys). In this direction, Separovic et al. [31] proposed that the additional residues in maculatin with respect to aurein not only increase the peptide length, in order to allow the total span of the bilayer, but also provide a smaller hydrophilic cylindrical surface area in the helix. The difference in peptide length could be an important factor in the lytic mechanism [12]. Aurein is long enough to form a well-defined amphipathic helix, although it is not capable of spanning across the POPC bilayer. At least 22 amino acids are required to extend an α-helix across the anisotropic axis of a bilayer [12]. Maculatin fits this requirement. However, when aggregated inside the bilayer, maculatin peptides were found at multiple conformations ranging from a U-shape to a completed extended one, mainly due to the kink in its structure.

All of the simulations carried out here exhibit two general characteristics of the peptides in terms of clustering tendency. Aurein peptides can adopt two different states: aggregated or isolated. The isolated population, as we described above, binds to the lipid–water interface with a well-defined orientation: non-polar and polar residues face the membrane and water, respectively. In contrast, isolated maculatin molecules were rarely observed, since the maculatin molecules showed a strong tendency to form aggregates between each other, especially in polar environments. In fact, practically all of the maculatins were involved in clusters. It is important to highlight that both peptides have a tendency to form aggregates in the water phase (see supplementary material). These results were also found using atomistic simulations (data not shown).

When maculatin is forced to be isolated (small systems with two peptides placed on opposite sides of the bilayer), it can bind to the interface with a defined orientation, corresponding to the polar and non-polar faces, in a similar way of aurein. Bond et al. [21] observed maculatin pore formation on POPC liposomes using MD simulations within the modified MARTINI force field [32]. Maculatin molecules penetrated the bilayer only as part of clusters, while individual molecules remained bound at the interface. On the other hand, Shahmiri [33] proposed a mechanism for aurein that involves an early aggregation of peptides upon a threshold prior to membrane penetration. According to this vision, aurein does not act by removing lipid molecules individually, but rather by changing the collective properties of the membrane. The need of peptide aggregation is coincident with all of the simulations carried out here. Furthermore, no pore formation was observed when the lipid–lipid proximity is favored (aurein *self-C* case).

We found that aurein peptides were able to adopt organized structures in a pore-like shape, which was observed by the analysis of terminal amino acids and their tilt angle. The hydrophobic residues faced the lipid tails and formed a structure with a polar interior, which allowed water transport through it. The establishment of a well-formed hydrophilic channel is facilitated by the equilibrated distribution between polar and non-polar residues in aurein. In contrast, maculatin has a larger non-polar surface, so despite the factor that maculatin has the same hydrophobic moment and net charge than aurein, the hydrophilic character of the channel is visibly reduced; thus, the permeation of water across the pore is lower. As we already mentioned, aurein pore-like structures are more organized than the maculatin ones. It is important to point out here that the size of a CG water molecule precludes us to assert this result. In this direction, atomistic simulations would be advisable to shed light on the permeation mechanism. As pointed out by Bennett et al. [34], polarizable water improves the free energies, but not the structure, to the same extent.

Our results show the specific orientation of aurein terminals inside the membrane: The NT terminals are found in the proximity of the lipid–water interface, while the CT terminals localized in the hydrophobic core of the bilayer. Even further, the aurein orientation is accompanied by a specific tilt angle formed by the CT to NT vector ranging from 20°–25° with the plane of the bilayer. In agreement with these results, the tilt angle of aurein inserted into DMPC bilayers is nearly 30°, as estimated by circular dichrosim (CD) [35]. By contrast, maculatin does not show this kind of organization with a preferential tilt angle.

In this way, while aurein molecules conforming to pore-like structures follow well-defined orientation patterns, maculatin peptides seem to act via a crowding behavior driven by their more hydrophobic character. In accordance with Bond [21], our results show that maculatin aggregates are not well-defined pores, which also exclude the lipid head groups from structures. Furthermore, the aggregates form neither barrel-stave or toroidal kinds of pores.

Aurein *-in* and *-self* simulations display two well-defined populations, isolated or aggregated, with a number of molecules involved in pore structures varying between 10 and 24. On the other hand, maculatin molecules are rarely isolated in water or at the water–lipid interface; rather, they aggregate in clusters. For example, in a *self-A* simulation, where maculatin molecules were distributed equidistant, five aggregates were observed.

We also evaluated the concentration effects of maculatin in the cluster formation. At a 50:1000 peptide–lipid ratio, most of the maculatin peptides are involved in one large cluster, which crosses the membrane and extends to the aqueous phase. Decreasing the initial number of peptides from 50 to 20 molecules leads to the formation of two aggregates instead of one. Furthermore, comparing the self-assembly cases, we found a higher number of cluster structures when the peptides were homogeneously distributed (maculatin *self-A* case). Experimental reports [27] show that maculatin reaches 100% POPC membrane leakage with a peptide–lipid ratio 10 times lower than aurein.

In the same line, when the peptides were initially placed in water in the presence of a bilayer, a clear-cut difference between aurein and maculatin behavior was observed. There is no evidence that aurein causes structural alterations on the lipid structure when originally placed in water. It is absorbed at the interface. However, we observed an influence of maculatin over the membrane structure in terms of curvature. These data are in line with the differences that we found from the self-assembly simulations. Maculatin shows cluster formation together with lipid structures that have a cylindrical symmetry. On the other hand, no evidence of a curvature-induction effect was observed for aurein clusters or individual aurein molecules. Changes in local curvature could be associated with pore formation, as reported for the helical AMP magainin [36], and simple sequences, such as hexarginin (R6), are capable of inducing pore-like structures and permeating membranes based on induced curvature [37,38]. Furthermore, the maculatin curvature effect is somehow comparable with the toroidal type of pores [36], where lipid head groups are forced to stay in a highly-curved region in order to connect the two leaflets of the membrane. Previous MD simulations of POPC liposomes [21] also show the maculatin curvature-induced effect: a cluster insertion resulted in a more ellipsoidal shape, and led to a marked increase in the membrane tension.

## 3. Methods

### 3.1. Coarse-Grain Model

MD simulations were performed with the GROMACS 4.5.5 software package [39,40,41,42]. The four-to-one MARTINI [43,44] mapping was chosen, using the improved parameters published by de Jong et al. [45] for the amino acids, in combination with the polarizable water (PW) model [46]. To capture the inhomogeneous nature of the dielectric response, CG water was used. This model consists of three particles: a central particle (neutral, which interacts with other particles by Lennard–Jones interactions), and two additional particles bound to the central particle, which carries a positive and negative charge. The main reason for having included polarizability into the model is the expectation that processes involving interactions between charged and polar groups in a low-dielectric medium are more realistically described [46]. In this direction, a dielectric constant of 2.5 was considered.

The tridimensional structures of aurein 1.2 and maculatin 1.1 were taken from the Protein Data Bank [47] codes 1VM5 [48] and 2MMJ [48]. The 1VM5 structure was used for aurein, while the 2MMJ was modified with two punctual mutations (GLY-to-PRO and I4G-to-ALA) in order to obtain the wild-type maculatin molecule. I4G corresponds to *n*-(2-methylpropyl)glycine present in the NMR structure. These structures were also used for the calculation of the secondary structure, since the MARTINI force field needs a secondary structure assignment Define Secondary Structure of Proteins (DSSP) file [49,50] as input. Even if the secondary structure is fixed—and this could be a limitation to study peptides in different environment, i.e., the water phase—our main goal here is to sample them in the lipid phase in order to obtain insights on their collective behavior. 

In this way, the CG simulations were carried out with the minimum degree of restraints beyond the MARTINI classical secondary structure ones. The C-terminus of both aurein and maculatin are naturally amidated, so both C-terminal beads was considered with charge zero, instead of the default negatively-charged C terminal in the MARTINI model. Chloride and sodium counter ions were added using the genion tool provided with the GROMACS package to each system, in order to equilibrate the net charge and to emulate ~0.15 M of NaCl buffer.

### 3.2. Sequence and Structure Analysis Tools

The calculation of the mean hydrophilicity and percentage of hydrophilic residues was performed with the online calculator of peptide properties provided by BACHEM [51]. Mean hydrophilicity was estimated by using the Hopp and Wood’s scale. This scale assigns a numerical value to each amino acid based on hydrophilicity. The non-polar residues have negative values [28].

The calculation of the hydrophobic moment was performed with the *Totalizer* tool of the Membrane Protein Explorer (MPEx) 3.2.15, which was developed by the laboratory of Dr. Stephen H. White [52]. To determine the hydrophobic moment [53] μH, *Totalizer* computes the modulus of the mean vector sum of the hydrophobicity vector **H** for each individual amino acid, using the Wimley–White interfacial hydrophobicity scale [54]. The helical wheel projections were created using the online application *NetWheels* [55].

The pairwise sequence alignment was done with the *EMBOSS water* [56] tool. *EMBOSS water* is an implementation of the Smith–Waterman algorithm [57]. Alignment was done with a BLOSUM62 matrix working with a gap penalty of 10.0 and an extend penalty of 0.05.

### 3.3. MD Simulation Conditions

MD simulations were performed with a time step of 20 fs and Coulomb/van der Waals distances cutoff of 1.2 nm, in a NP_XY_P_Z_T ensemble, using periodic boundary conditions (PBC). The reference temperature was coupled at 323 K with a Berendsen thermostat at a time constant of 1.0 ps. The pressure was coupled with a semi-isotropic barostat (xy and z pressures were coupled independently at 1 bar). Equilibration was carried out, taking into account the initial conditions, followed by a simulation production run.

## 4. Conclusions

In this study, we used coarse grain molecular dynamic simulations to monitor the interactions of two antimicrobial peptides with POPC lipid structures at the molecular level. We studied the systems from very different initial conditions in order to get a good perspective of the possible structures that the antimicrobial peptides could reach in a lipid environment. Our simulations were able to capture interesting differences between aurein and maculatin behavior that could guide experimental interpretation. Aurein could form organized pore-like structures that allow water transport (even at the CG level). On the other hand, the maculatin unorganized clusterization and curvature induction effects in bilayers seem to be characteristics that could be responsible for membrane destabilization (even leading to lytic activity).

The MARTINI coarse grain model used here, as with most simplified models, has its limitations, and it is important to account for them when drawing conclusions [45]. For instance, the four-to-one mapping reduces the chemical resolution, the thermodynamic properties, such as the balance between enthalpy and entropy, are particularly affected, and the capability to pore formation is limited. This last point could be partially attributed to the CG models’ high bending modulus, which was shown to be nearly double atomistic and experimental values [34]. Besides, in the framework of this work, we would like to point out the protein (and peptide) backbone design features to the secondary structure in MARTINI forcefield. The used constraints maintain the peptides helical secondary structure that corresponds to the membrane environment. Within this approach, the description of peptides in water phase would become less reliable. Even with these limitations, the CG model is successful in many cases when compared to atomistic and experimental results.

One useful application of the MARTINI CG model that we took advantage of here is to size up the structures sampling that is required to study a particular phenomenon [45]. Here, we centered our discussion on structural more than dynamical properties, thus avoiding the CG time scale interpretation discussion. In this direction, the obtained results pointed out important features on the collective effects of aurein and maculatin in lipid structures, which are difficult to access through other techniques. This could guide further exploration, such as through *backmapping* combined with atomistic simulations, for instance. 

## Figures and Tables

**Table 1:** Summary of simulations.

Case	Description	System Configuration	Final Box Size	Time
Out	Outside bilayer, large	1000 POPC/50 peptides/ 22,639 PW/50 NA/100 CL	18.55 × 18.55 × 12.13 18.56 × 18.56 × 12.25	2 μs (aurein 1.2) 3 μs (maculatin 1.1)
Small-out	Outside bilayer, avoiding aggregation	128 POPC/2 peptides/ PW/NA/CL	6.50 × 6.50 × 12.43 6.50 × 6.50 × 12.46	2 μs
In-A	Inside bilayer, big	1000 POPC/50 peptides/ 22,639 PW/50 NA/100 CL	18.77 × 18.77 × 11.81 19.10 × 19.10 × 11.56	1 μs
In-B	Inside the bilayer large, low P:L ratio	1000 POPC/20 peptides/ 22,639 PW/50 NA/70 CL	18.58 × 18.58 × 11.80 (only maculatin 1.1)	1 μs
Small-in	Inside the bilayer small	128 POPC/8 Peptides/ 2976 PW/8 NA/16 CL	6.84 × 7.29 × 10.82 6.86 × 7.32 × 10.89	4 μs
Self (A, B, and C)	Large self-assembly	1000 POPC/50 Peptides/ 22,639 PW/50 NA/100 CL	13,09 × 13,09 × 24.31 14.54 × 14.54 × 19.70	1 μs

## Supplementary Materials

The following are available online. Figure S1: Small-in aurein system and the pore-like structure. Snapshot from a frontal plane, showing the presence of water molecules inside the interior channel, Figure S2: Small-in maculatin system and the pore structure. Snapshot over XY bilayer plane showing a small hydrophilic surface at the interior of the cluster structure, Figure S3: Electron density profile (EDP) system profile of maculatin-in case. Amino terminals (blue) and carboxylic terminals (green) show preferential position along the *z*-axis. Water and POPC molecules are depicted in black and red, respectively, Figure S4: Simulations of aurein 1.2 (A) and maculatin 1.1 (B) in water. Both peptides exhibit a high clustering tendency.
