# Supplementary figures and images for: Differential Interaction of Antimicrobial Peptides with Lipid Structures Studied by Coarse-Grained Molecular Dynamics Simulations
Source: Molecules. 2017 Oct 20;22(10):1775. doi: 10.3390/molecules22101775 (PMC6151434; doi:10.3390/molecules22101775)

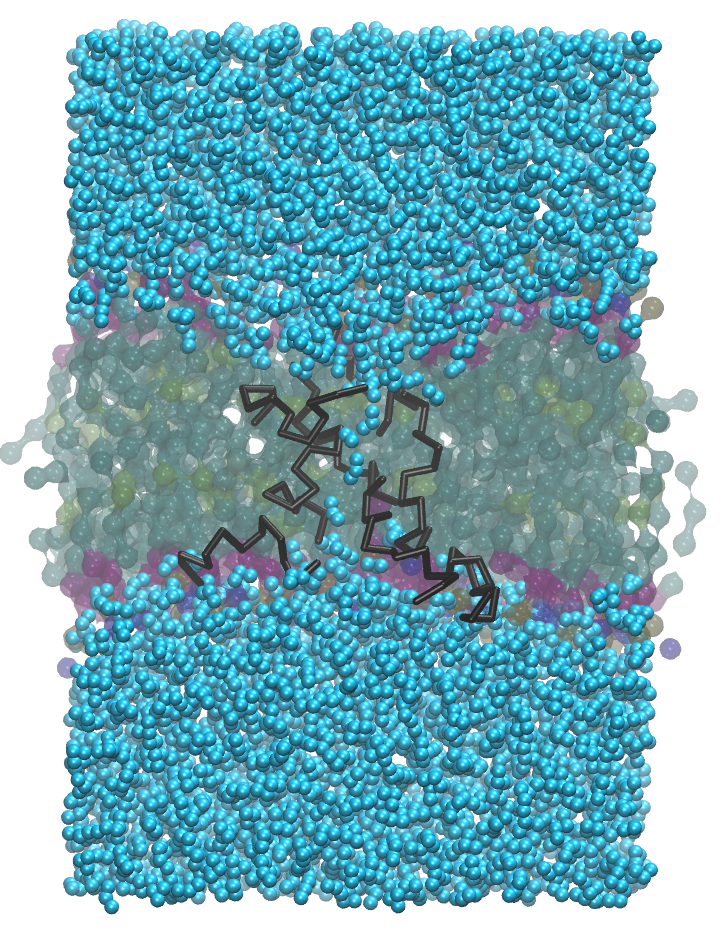

Supplement: Supplementary file 1 [file molecules-22-01775-s001.zip › Supplementary/sup1.tif]

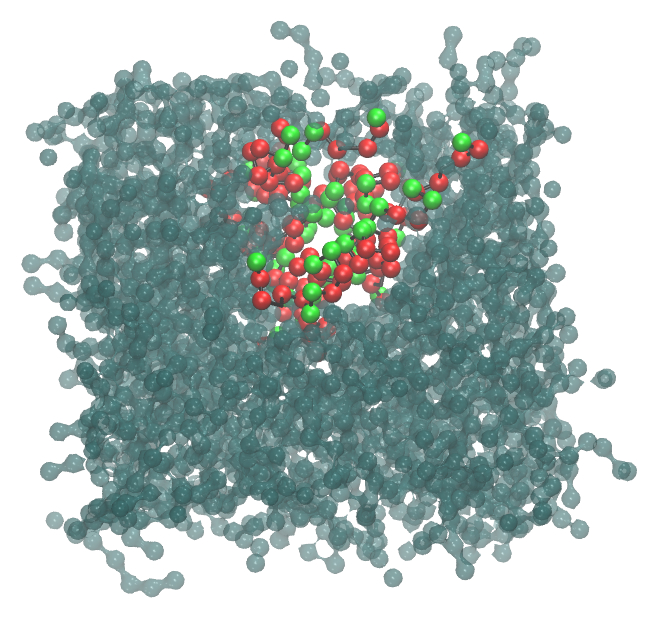

Supplement: Supplementary file 1 [file molecules-22-01775-s001.zip › Supplementary/sup2.tif]

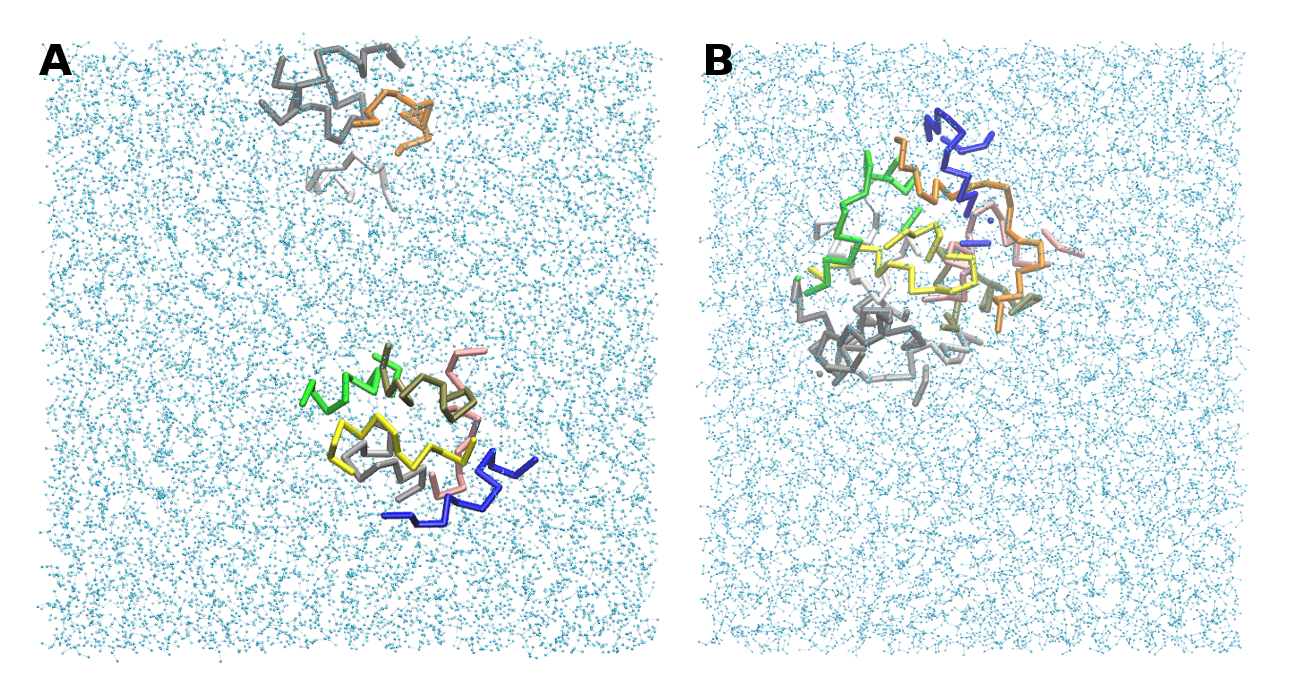

Supplement: Supplementary file 1 [file molecules-22-01775-s001.zip › Supplementary/sup4.tif]
